# A Study of Food Safety Knowledge for Sustainable Foodservice Management of Childcare Centers in South Korea Using Importance–Performance Analysis

**DOI:** 10.3390/ijerph19159668

**Published:** 2022-08-05

**Authors:** Jeong-Sil Choi, Se-Young Ju

**Affiliations:** Department of Food Science, College of Biomedical and Health Science, Konkuk University, Chungju 27478, Chungbuk, Korea

**Keywords:** food safety, employees, childcare center, importance, performance, sustainable foodservice management, IPA

## Abstract

This study aims to evaluate the importance and performance level of knowledge about sanitary management among foodservice employees in childcare centers that were registered at Center for Children’s Food Service Management in Chungju city according to their work duration, type of childcare center, and number of enrolled children. The self-administered questionnaire was conducted to examine food safety attributes of sanitary management at 150 childcare centers without qualified dietitians registered at Center for Children’s Food Service Management of Chungju city. The questionnaire consisted of 15 questions about perceived importance and performance regarding sanitation management (personal hygiene, ingredient control, temperature control of food, facility, equipment, and utensils sanitation) using IPA (importance–performance analysis). The results show that overall mean scores of the importance and performance of sanitary knowledge were 4.71 and 4.67 out of 5, respectively. ‘Checking the center temperature at 75 °C for 1 min in the thickest part of meat (3 times or more check for each serving)’ (*p* = 0.047) and ‘Keeping preserved meals (at least 100 g of each menu) for 144 h. with −18 °C or less’ (*p* < 0.001) show significantly lower scores of performance than those of importance. The results of importance and performance for sanitary management according to work duration of foodservice employees show that those who have worked more than 10 years had the highest scores of importance and performance for overall sanitary management among them. For the types of childcare centers, the overall performance scores of national/public employees for sanitary management were lower than those of private or home type (*p* < 0.001). Additionally, the result showed that the overall importance (*p* < 0.001) and performance scores (*p* < 0.001) of employees for sanitary management in centers with <50 children were higher than those in centers with ≥50 children. This result should provide more useful information to develop food safety programs for employees and sustainable foodservice management in childcare centers.

## 1. Introduction

Children in childcares have become common throughout our society according to the increase in dual-income families and downsized family members. A total of 35,352 childcare centers caring for 1,244,396 children under age 6 were reported nationwide in 2021 [1]. Due to the increased use of childcare centers and the longer staying times in out-of-home childcare, children attending childcare centers are generally provided with at least one meal and snacks daily. Therefore, the nutrition and hygiene management of childcare food service is a necessary and important point as the demands of high dietary quality in childcare are increasing.

In the Korean Food Sanitation Act (2021), food service facilities with less than 50 persons do not have to follow sanitary inspection obligations of local governments and childcare centers with less than 100 children are not obligated to hire a registered dietitian. A total of 79% of childcare centers in Korea have less than 100 children, of which 70% have 50 children or less. The majority of childcare centers have difficulties in operating proper nutrition and hygiene management for children. There are several research reported on the poor-quality food service from the lack of registered cooks [2], deficient recognition of sanitary management [3], and lack of necessary food service facilities and equipment [4,5]. Because of these problems and social requirements, The Ministry of Food and Drug Safety enacted the “Special act on Food Safety Management for Children” in 2008 and is operating a Center for Children’s Food Service Management (CCFSM), which supports the foodservice management of hygiene, safety, and nutrition provided by childcare centers without a registered dietitian. With the establishment of 12 centers in 2011, 229 centers were operating nationwide in 2020. The main roles of the Center for Children’s Food Service Management are regularly visiting those childcare centers to guide sanitary and nutrition foodservice management, educate children, parents, employees, provide menu and recipes, and the provision of information and education programs that are related to foodservice [6]. These efforts are showing positive results through quite a few studies, such as the improvement of children’s diet behaviors [7] and positive effects of a periodic visiting on education programs for employees and parents of sanitary and safety management [5,8,9,10,11]. The importance–performance analysis (IPA) has been developed for measuring the elements of marketing programs because of a simple and easy understanding technique [12,13]. Due to these advantages, it is applied in various fields, such as tourism [14,15,16], foodservice [17,18,19,20], education [21,22], healthcare [23,24], and public administration [25].

The objective of this study is to evaluate the importance and performance level of knowledge about sanitary management among foodservice employees in childcare centers that were registered in Center for Children’s Food Service Management in Chungju city according to their work duration, type of childcare center, and number of enrolled children. The results could be used to determine the attributes to be improved first in the sanitary management of childcare centers. Furthermore, the results of this study can be the base of more useful programs of sanitary management for childcare centers and operate more sustainable foodservice management of childcare centers.

## 2. Methods

### 2.1. Subjects and Questionnaire Design

A survey was conducted to examine the cooks’ attributes of sanitary management at 150 childcare centers without qualified dietitians registered at CCFSM (Center for Children’s Food Service Management) of Chungju city. The self-administered questionnaire was distributed among registered childcare centers at Center for Children’s Food Service Management of Chungju city between May and August of 2020. A total of 150 questionnaires were collected, and all were used for analysis.

The questionnaire was developed based on the sanitary checklist according to the guidelines of Center for Children’s Food Service Management from Ministry of Food and Drug Safety [26]. The questionnaire was pretested by 30 randomly selected food handlers in childcare centers and examined by 3 food safety experts. The questionnaire was modified by the feedback of the results. Therefore, the questionnaire was approved for ensuring reliability and validity. The questionnaire contained two parts. The first part consisted of 15 questions about perceived importance and performance regarding sanitation management (personal hygiene, ingredient control, temperature control of food, facility, equipment, and utensil sanitation) using IPA (importance–performance analysis). The second part consisted of questions on the general characteristics of the respondents (age, work period, certificate, and daily working hours) and childcare centers (types and number of enrolled children). Regarding the importance and performance of sanitation management, it was measured on a five-point Likert scale, with answers range from 1 (not very important, not performed very much) to 5 (very important, performed very much).

### 2.2. Data Analysis

IPA is a simple technique for data analysis, which measures people’s importance and performance about attributes and identifies those attributes that need improvement the most. Additionally, the results of the IPA can be categorized in a two-dimensional IPA grid that displays the results graphically. The quadrants were divided according to the average of important and performant scores. The results of 15 attributes were positioned in four quadrants: Keep up the good will (Quadrant I), which is perceived to be very important to respondents and has high levels of performance; Concentrate here (Quadrant II), which is perceived to be very important to respondents, but has fairly low levels of performance, so these attributes need improvement efforts; Lower priority (Quadrant III), which has low levels of importance and performance; and Possible over kill (Quadrant IV), which is perceived to be low important, but has relatively high levels of performance. The results were analyzed for diagnosing the difference between perceived importance and performance of knowledge about sanitary management [12,13]. This study reviewed and approved by OO University Institutional Review Board (IRB approval number: 7001355-202204-HR-536).

### 2.3. Statistical Analysis

All analyses were performed using SPSS program version 26.0. The general characteristics of respondents and those of the childcare centers were assessed through frequency and descriptive analyses. The difference between the importance and performance of sanitation management knowledge was analyzed with a paired t-test, while each importance and performance of sanitation-related knowledge according to age, work period, and type of childcare center was assessed using ANOVA and Duncan’s multiple-range tests with a significant level of *p* < 0.05. Each importance and performance grid analysis of sanitation-related knowledge by age, work period, and type of childcare center was assessed using the mean value of importance and performance to mark the performance as the *x*-axis and the importance as the *y*-axis.

## 3. Results

### 3.1. General Characteristics

The general characteristics of the respondents and those of the childcare centers in Chungju are presented in Table S1. Those in their 50 s accounted for the majority with 42.7%, followed by 33.4% in their <40 s and 23.3% in their ≥60 s. A total of 52.0% of foodservice employees has worked for less than 5 years in foodservice facilities, while 26.0% of the respondents have worked in the sector for more than 10 years. Cooks with a certificate accounted for 95.3% of the respondents. More than 80% provided at least a mid-morning snack, lunch, and an afternoon snack. As for the average working hours per day, foodservice employees mainly worked for 4–8 h per day (74.7%).

Regarding the type of childcare centers where the respondents worked, 48.7% were operated in the form of private childcare centers, followed by national and public types (15.3%) and the home type (18.0%). The percentage of enrolled children was 34.7% (aged between 1 and 2) and 30.1% (aged between 3 and 4), accounting for more than 50%. A total of 72.7% of childcare centers had less than 50 children enrolled, followed by 27.3% with less than 100 children.

### 3.2. Importance and Performance Analysis of Sanitary Knowledge

Table 1 shows the importance–performance evaluation of knowledge related to sanitation at childcare centers in Chungju. The overall mean score of performance was 4.67, which is lower than that of the importance score (4.71) (*p* < 0.001). The perceived importance of sanitation-related knowledge and the perceived sanitary practices of foodservice management were generally well known to all respondents. Among the 15 questions addressing sanitary foodservice management, ‘Excluded from work if you have symptoms of vomiting or diarrhea, or wounds on your hands’ (Personal hygiene 1) and ‘Using food ingredients First-in First-out method’ (Ingredient control 4) had the highest scores of 4.74 out of 5. In the performance score, ‘Ingredient control 4’ and ‘Serving cooked food within 2 h’ (Temperature control of food 2) had the highest scores of 4.76 out of 5.

On the other hand, regarding the perceived importance of sanitary practices, ‘Monitoring the temperature of the refrigerator (5 °C or less) and the freezer (−18 °C or less) at least twice a day’ (Equipment and utensils sanitation 1) and ‘Fresh vegetables and fruits without cooking should be disinfected in chlorine within 5 min and rinsed 3 times or more’ (Ingredient control 1) showed the lowest scores of 4.64 and 4.65 out of 5. Regarding performance, ‘Keeping preserved foods (at least 100 g of each menu) for 144 h with −18 °C or less’ (Temperature control of food 3) had the lowest score of 4.34, followed by ‘Equipment and utensils sanitation 1’ (4.55), ‘Ingredient control 1’ (4.60), and ‘Checking the center temperature (75 °C for 1 min) of the thickest part of meat (3 times or more for each serving)’ (Temperature control of food 1) (4.62).

As a result of the performance analysis according to importance among all 15 questions, it was found that the performance was significantly low compared to the importance ‘Temperature control of food 1’ (*p* = 0.047) and ‘Temperature control of food 3’ (*p* < 0.001).

### 3.3. Analysis of Importance–Performance for Sanitation-Related Knowledge

**Figure 1:**
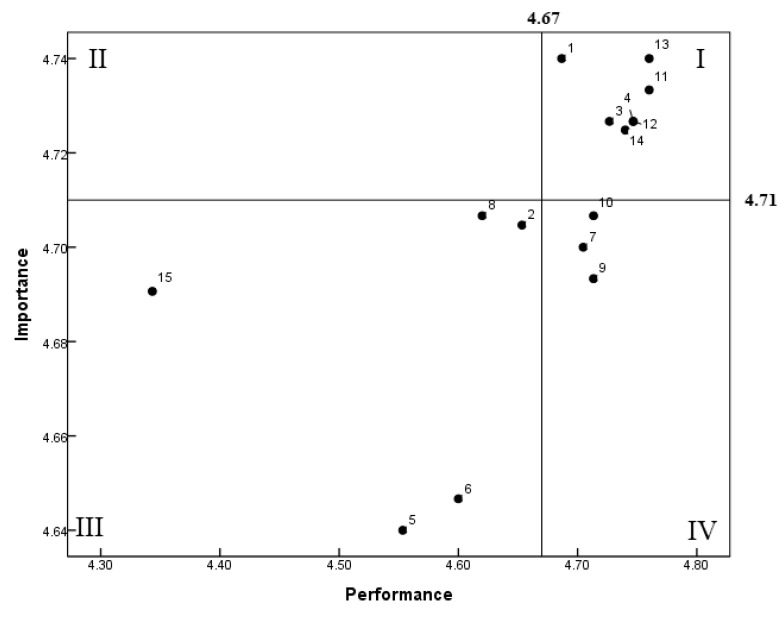
Importance–performance analysis (IPA) matrix about sanitary knowledge of foodservice management at childcare centers in Chungju. 1: Personal hygiene 1; 2: Personal hygiene 2; 3: Personal hygiene 3; 4: Personal hygiene 4; 5: Equipment and utensil sanitation 1; 6: Ingredient control 1; 7: Ingredient control 2; 8: Temperature control of food 1; 9: Ingredient control 3; 10: Equipment and utensil sanitation 2; 11: Temperature control of food 2; 12: Equipment and utensil sanitation 3; 13: Ingredient control 4; 14: Ingredient control 5; 15: Temperature control of food 3.

IPA analysis is shown in Figure 1. Quadrant I, whose attributes are perceived to be very important and have high levels of performance, corresponds to questions 1 (Personal hygiene 1), 3 (‘Wash and disinfect hands before working and change process’, Personal hygiene 3), 4 (‘Medical check-up should be conducted at least once a year’, Personal hygiene 4), 11 (Temperature control of food 2), 12 (‘Washing and sterilizing kitchen equipment and utensils after use’, Equipment and utensils sanitation 3), 13 (Ingredient control 4), and 14 (‘Do not store expired raw materials and products for the purpose of cooking’, Ingredient control 5), which require continuous management with high importance and performance. Quadrant II is an area with high importance but low performance that requires intensive management (20), but none of the aspects in this area were applicable. This shows that sanitation-related knowledge is important and that it is being followed. Quadrant III is an area of low importance and low performance. ‘Personal hygiene 2’ (Wear clean uniform, hair cap, and apron without accessories or nail polish), ‘Equipment and utensils sanitation 1’, ‘Ingredient control 1’, ‘Temperature control of food 1’, and ‘Temperature control of food 3’ were applicable to this area.

### 3.4. Analysis of Importance–Performance by Work Period

**Figure 2:**
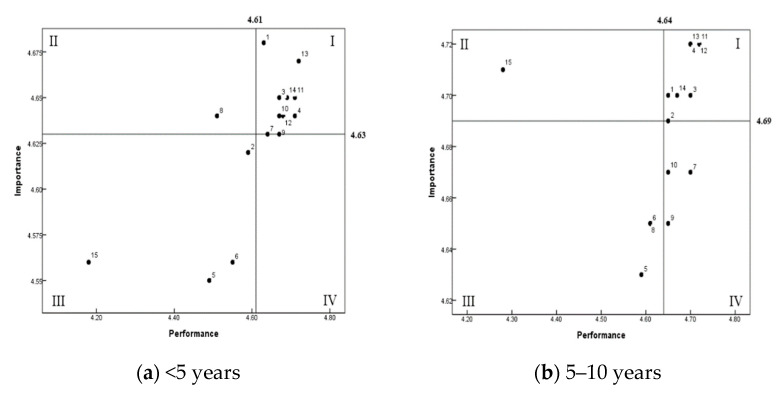

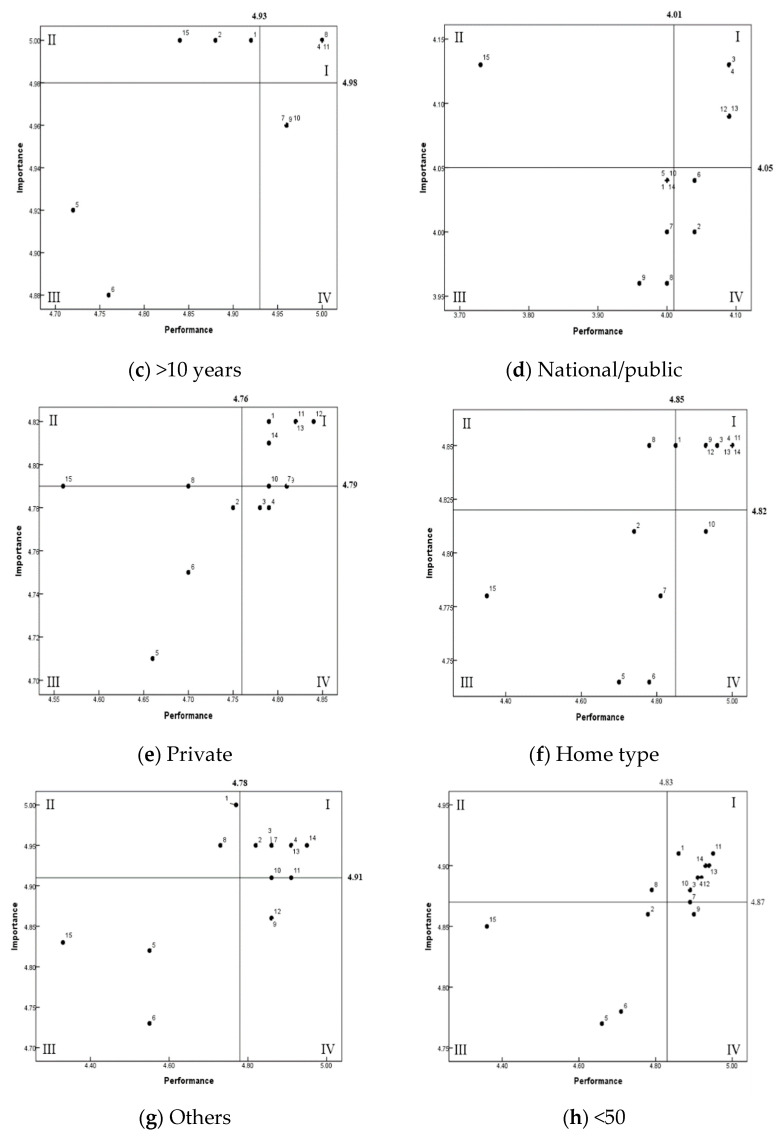

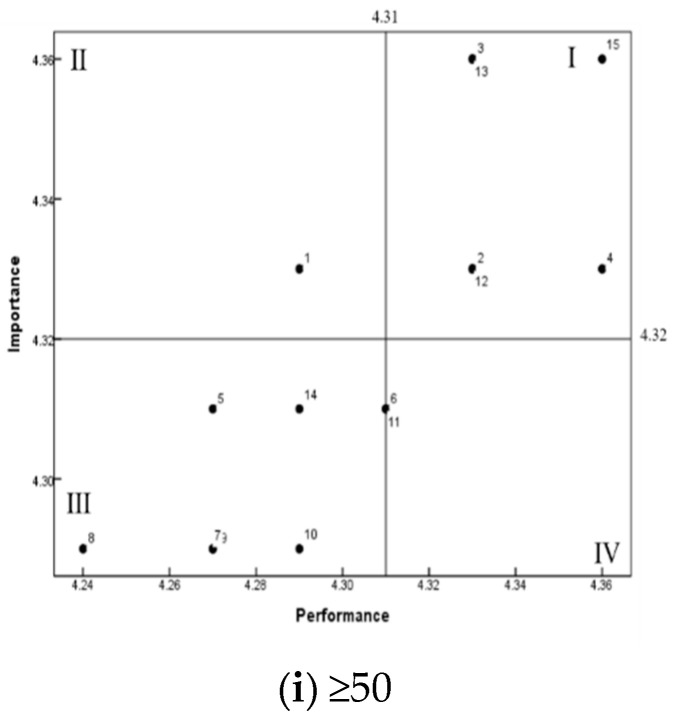
Importance–performance analysis (IPA) matrix about sanitary knowledge of foodservice management at childcare centers in Chungju by work period (years) (**a**–**c**), type of childcare centers (**d**–**g**), and number of enrolled children (**h**,**i**). 1: Personal hygiene 1; 2: Personal hygiene 2; 3: Personal hygiene 3; 4: Personal hygiene 4; 5: Equipment and utensil sanitation 1; 6: Ingredient control 1; 7: Ingredient control 2; 8: Temperature control of food 1; 9: Ingredient control 3; 10: Equipment and utensil sanitation 2; 11: Temperature control of food 2; 12: Equipment and utensil sanitation 3; 13: Ingredient control 4; 14: Ingredient control 5; 15: Temperature control of food 3.

Table 2 shows the importance–performance analysis regarding knowledge related to sanitation according to the work period of foodservice employees. The overall mean scores of importance and performance of sanitary knowledge with more than 10 years of work were significantly the highest among the respondents (less than 5 years, from 5 to 10 years, and more than 10 years). The foodservice employees with more than 10 years of working experience had better sanitary foodservice knowledge and performed sanitary foodservice management practices better than the other respondents. Figure 2 shows the result of the grid analysis for importance and performance by work period. The quadrant II area, which means high importance and low performance, needs to be improved as a priority; one question, ‘Temperature control of food 1’, belongs to this area in the group with less than 5 years. Additionally, ‘Temperature control of food 3’ belongs to this area in the group of work period from 5 to 10 years. In addition, ‘Personal hygiene 1, ‘Personal hygiene 2’, and ‘Temperature control of food 3’ correspond to the group of work period more than 10 years.

### 3.5. Analysis of Importance–Performance by Types of Childcare Center

Table 3 and Figure 2 show the importance–performance analysis regarding knowledge related to sanitation according to types of childcare centers. The types of childcare centers were divided into four groups: national/public, private, home type, and others. The overall mean score of importance and performance about sanitary knowledge of foodservice management was significantly lower in the foodservice employees of the national/public type than that of the other groups (private, home type, and others) (*p* < 0.001). There were significant differences among the groups, except for the performance item ‘Temperature control of food’ 3 (*p* = 0.059). In the importance–performance analysis matrix (Figure 2) for the group of national/public, ‘Temperature control of food 3’ was applicable to the quadrant II. For the home type group, ‘Temperature control of food 3’ belongs to the quadrant II, while two items, ‘Temperature control of food 3’ and ‘Personal hygiene 1’, correspond to the others group.

### 3.6. Analysis of Importance–Performance by the Number of Enrolled Children in the Childcare Centers

Table 4 and Figure 2 present the results of the IPA regarding sanitary knowledge and practices according to the number of enrolled children in childcare centers. The overall mean scores of importance and performance for childcare centers with <50 enrolled children (4.87 and 4.83, respectively) were significantly higher than those for childcare centers with ≥50 enrolled children (4.32 and 4.31, respectively) (*p* < 0.001). The employees of childcare centers with <50 enrolled children showed higher perceived importance levels about the knowledge of personal hygiene, ingredient control, temperature control of food, and equipment and utensil sanitation, and showed a similar tendency of performance levels about the sanitary practices of foodservice management. On the other hand, those with ≥50 enrolled children showed relative importance and performance scores about personal hygiene (mean scores = 4.34 and 4.33, respectively), ingredient control (mean scores = 4.31 and 4.29), temperature control of food (mean scores = 4.32 and 4.30), and equipment and utensil sanitation (mean scores = 4.31 and 4.30). In Figure 2, in the quadrant II area of that which needs to be improved as a priority, one item, ‘Temperature control of food 1’, corresponds to this area for the group of childcare centers with <50 enrolled children. For the group of those with ≥50 enrolled children, one item, ‘Personal hygiene 1’, belongs to the quadrant II area.

## 4. Discussion

This study examined the perceived importance and performance levels of knowledge about sanitary management among foodservice employees in 150 childcare centers that are registered in the Center for Children’s Food Service Management in Chungju city and investigated the differences in the importance and performance levels of sanitary knowledge according to their work duration, type of childcare center, and number of enrolled children.

The Center for Children’s Food Service Management (CCFSM) operates for childcare centers with less than 100 children that were not obligated to hire a registered dietitian and supports the foodservice management of hygiene, safety, and nutrition and education programs for the children, parents, and employees of those childcare centers. The results show that the overall mean scores of the importance and performance of sanitary knowledge were 4.71 and 4.67 out of 5, respectively. The overall mean score of importance was higher than that of performance, even if those were not significantly different (*p* = 0.242). However, the mean scores of importance and performance about ‘Temperature control of food’ were significantly different as 4.71 and 4.57, respectively (*p* = 0.037). Especially, ‘Checking the center temperature (75 °C for 1 min) of the thickest part of meat (3 times or more check for each serving)’ (*p* = 0.047) and ‘Keeping preserved meals (at least 100 g of each menu) for 144 h with −18 °C or less’ (*p* < 0.001) showed significantly lower scores of performance than those of importance. Very young people belong to one of the groups that are highly susceptible to food-borne illnesses and children in childcare centers are especially vulnerable to foodborne illness outbreaks. According to the recent statistics of Korea, schools had the highest number of foodborne illness outbreaks, followed by those of companies, kindergartens, and childcare centers (MFDS, 2018).

Many foodborne illness outbreaks are caused by inadequate cooking and improper handling of food. Measuring temperatures of potentially hazardous food, especially food items containing protein and moisture and requiring time and temperature control to prevent the growth of microorganisms, is an important responsibility of foodservice employees (Food code, 2009). Therefore, ‘Checking the center temperature (75 °C for 1 min) of the thickest part of meat (3 times or more check for each serving)’ is one of the focal points for preventing foodborne illnesses in young children.

The results of the importance and performance analysis for sanitary management according to the work duration of foodservice employees show that those who have worked for more than 10 years had the highest scores of importance and performance for overall sanitary management. However, in the IPA grid, the foodservice employees who have worked for more than 10 years had three questions of sanitary management (2 personal hygiene and 1 temperature control of food) with high importance and low performance (quadrant II) and those with 5~10 years and those with less than 5 years had one question with that area. Those with >10 year of work duration had especially low performance levels of personal hygiene, which are ‘Excluded from work if you have symptoms of vomiting or diarrhea, or wounds on your hands’ and ‘Wear clean uniform, hair cap, and apron without accessories or nail polish’. The personal hygiene of foodservice establishments is the most basic requirement to be observed for employees. According to the World Health Organization (WHO), human actions are major source of food contamination during food handling and food preparation in foodservice facilities [27,28].

The question of ‘Keeping preserved meals (at least 100 g of each menu) for 144 h with −18 °C or less’ was found to have high importance and low performance (improvement efforts should be concentrate here) scores in the groups with >10 year and 5~10 years of work duration. Kim et al. [5] reported that the effects of food safety management support CCFSM. They examined the status of hygiene and safety practices of childcare centers with 50~<100 enrolled children and those with <50 enrolled children. The results showed that ‘preserved food management (Keeping preserved meals (at least 100 g of each menu) for 144 h with −18 °C or less)’ was not properly carried out, as when the amount of food preservation (at least 100 g of each menu) is small, only some food is preserved, or there is no dedicated freezer for preserved food. Additionally, they reported that the childcare centers with <50 enrolled children did not particularly keep preservation food management practices at all because of the lack of legal obligations to manage food preservation. The Korean Food Sanitation Act (2021) requires that ‘one serving of food on all menus provided for preserved for 144 h with −18 °C or less in the foodservice facility served ≥50 people’ to be used as basic data to conduct epidemiological investigations in the event of foodborne illness outbreaks. This requirement is very important to determine the cause of foodborne illness. Additionally, previous studies have shown that the insufficient and inadequate food safety knowledge and skills of foodservice employees resulted in unsafe food handling practices that cause the spread of foodborne pathogens [29,30,31]. Therefore, there is a need for the regulatory education and training of foodservice employees on food safety.

The results show that the overall performance scores of national/public employees for sanitary management were lower than those of the private or home types according to the types of childcare centers (4.01 vs. 4.76 vs. 4.86, respectively, *p* < 0.001). These results are different from those of Paik et al. [10] and Park et al. [20]. Paik et al. [10] reported that national or public childcare centers were more highly evaluated regarding the food safety score than private centers or corporations. The same result was found by Park et al. [20], and the result showed that the employees of national and public centers had higher importance and performance scores of sanitary management than those from private and home type childcare centers. Since private and home type childcare centers are usually managed by private owners, they should thoroughly educate and train foodservice employees on food safety and hygiene measures to prevent foodborne illnesses. In the IPA matrix, the questions located in quadrant II were ‘Keeping preserved meals (at least 100 g of each menu) for 144 h with −18 °C or less’ for the national/public childcare center and ‘Checking the center temperature (75 °C for 1 min) of the thickest part of meat (3 times or more check for each serving)’ for the home type childcare center.

For the number of enrolled children in childcare centers, overall importance (4.87 vs. 4.32, respectively, *p* < 0.001) and performance scores (4.83 vs. 4.31, respectively, *p* < 0.001) of employees for sanitary management in centers with <50 children were higher than those in centers with ≥50 children. Since Chungju city is a sparsely populated city, there are many small childcare centers (<50 children). In addition, those childcare centers would be well supported by the Center for Children’s Food Service Management in Chungju city.

The results of the IPA matrix show that the employees of centers with <50 children did not perform well on the question ‘Checking the center temperature (75 °C for 1 min) of the thickest part of meat (3 times or more check for each serving)’. For the employees of centers with ≥50 children, the question ‘Excluded from work if you have symptoms of vomiting or diarrhea, or wounds on your hands’ was located in quadrant II (improvement efforts should concentrate here). These two questions (temperature control and personal hygiene) are very important and are critical factors that cause foodborne outbreaks in foodservice facilities. Foodborne illnesses are globally playing a role in damaging public health and creating an economic burden [32,33]. Foodborne outbreaks are mostly caused by inappropriate food handling, such as cross contamination, improper storage condition, personal hygiene, and inadequate cooking in institutional foodservice establishments [34,35]. These causes are mainly caused by a lack of knowledge of basic food hygiene and safety of food-handling employees, as shown by previous studies [36,37,38,39]. In addition, since food handlers in small childcares are usually not registered and have no regular food safety education, they have food handling habits affected by their beliefs and norms [40]. Therefore, regulatory training and education of food safety for foodservice employees are necessary to operate effective sanitary management and improve foodservice quality in childcare centers. Training programs are suggested for regulatory training by visiting food safety experts of the CCFSM and using media training programs, such as videos and posters. Additionally, it is also necessary and effective that education programs of food safety knowledge are regularly conducted in the form of lectures from professional instructors.

There are several limitations to this study. First, because this survey was conducted by a voluntary and self-administered method, there might be the possibility that the evaluation was better than it really was. Because our sample size was small and included those registered in the Center for Children’s Food Service Management in Chungju city, it is difficult to generalize these results to all childcare centers. Second, this survey was conducted with only daycare centers registered with the CCFSM, which have non-registered dietitians. In future studies, comparative studies should be performed with childcare centers having registered dietitians. Third, because studies of childcare centers from other countries mainly focus on the microbiological assessment or observation of hygienic conditions [41,42,43], it is very unfortunate that we could not compare it with similar studies of other countries. However, this result offers basic information to provide education and training programs for foodservice employees and to operate more sustainable foodservice management in childcare centers.

## 5. Conclusions

The results of the overall mean score of importance were higher than those of performance at 4.71 and 4.67 out of 5, respectively. The questions, ‘Checking the center temperature (75 °C for 1 min) of the thickest part of meat (3 times or more check for each serving)’ (*p* = 0.047) and ‘Keeping preserved meals (at least 100 g of each menu) for 144 h with −18 °C or less’ (*p* < 0.001) showed significantly lower scores of performance than those of importance. The results of importance and performance for sanitary management according to the work duration of foodservice employees show that those who have worked for more than 10 years had the highest scores of importance and performance for overall sanitary management among them. For the types of childcare centers, the overall performance scores of national/public employees for sanitary management were lower than those of the private or home types (*p* < 0.001). The results show that the overall importance (*p* < 0.001) and performance scores (*p* < 0.001) of employees for sanitary management in centers with <50 children were higher than those in centers with ≥50 children.

## Figures and Tables

**Table 1:** Evaluation of the importance and performance of knowledge related to sanitation at childcare centers in Chungju (n = 150).

	Questions	Score		*p*-Value
		Importance (Mean ± SD)	Performance (Mean ± SD)	
Personal hygiene	1. Excluded from work if you have symptoms of vomiting or diarrhea, or wounds on your hands.	4.74 ± 0.97	4.69 ± 0.94	0.059
	2. Wear clean uniform, hair cap, and apron without accessories or nail polish.	4.70 ± 0.98	4.65 ± 0.95	0.158
	3. Wash and disinfect hands before working and change process.	4.73 ± 0.96	4.73 ± 0.90	0.999
	4. Medical check-up should be conducted at least once a year.	4.73 ± 0.96	4.75 ± 0.91	0.515
	Sub-total	4.72 ± 0.96	4.70 ± 0.92	0.697
Ingredient control	1. Fresh vegetables and fruits without cooking should be disinfected in chlorine within 5 min and rinsed 3 times or more.	4.65 ± 0.99	4.60 ± 0.96	0.162
	2. When thawing frozen food, use refrigerated thawing (5 °C or less), running water (21 °C or less), or microwave thawing, and do not refreeze thawed foods.	4.70 ± 0.98	4.70 ± 0.94	0.809
	3. Storing and handling foods and cooked foods at least 60 cm above the floor.	4.69 ± 0.97	4.71 ± 0.92	0.407
	4. Using food ingredients First-in First-out method.	4.74 ± 0.96	4.76 ± 0.91	0.493
	5. Do not store expired raw materials and products for the purpose of cooking.	4.72 ± 0.97	4.74 ± 0.92	0.639
	Sub-total	4.70 ± 0.97	4.70 ± 0.93	0.957
Temperature Control of food	1. Checking the center temperature (75 °C for 1 min) of the thickest part of meat (3 times or more for each serving).	4.71 ± 0.97	4.62 ± 0.99	0.047 *
	2. Serving cooked food within 2 h	4.73 ± 0.96	4.76 ± 0.91	0.319
	3. Keeping preserved foods (at least 100 g of each menu) for 144 h with −18 °C or less	4.69 ± 1.01	4.34 ± 1.24	<0.001 ***
	Sub-total	4.71 ± 0.97	4.57 ± 1.06	0.037 *
Equipment and utensil sanitation	1. Monitoring the temperature of the refrigerator (5 °C or less) and the freezer (−18 °C or less) at least twice a day.	4.64 ± 0.96	4.55 ± 0.97	0.063
	2. Separate use of knives, cutting boards, rubber gloves, and aprons.	4.71 ± 0.97	4.71 ± 0.92	0.783
	3. Washing and sterilizing kitchen equipment and utensils after use.	4.73 ± 0.96	4.75 ± 0.91	0.493
	Sub-total	4.69 ± 0.96	4.67 ± 0.94	0.752
Total		4.71 ± 0.97	4.67 ± 0.96	0.242

Note: *** *p* < 0.001, * *p* < 0.05.

**Table 2:** Importance and performance of knowledge related to sanitation at childcare centers in Chungju (n = 150) by work period.

Questions		Work Period and Score							
		Importance (Mean ± SD)			*p*-Value	Performance (Mean ± SD)			*p*-Value
		<5 Years	5–10	>10 Years		<5 Years	5–10	>10 Years	
Personal hygiene	1	4.68 ± 1.08	4.70 ± 1.03	5.00 ± 0.00	0.335	4.63 ± 1.03	4.65 ± 1.02	4.92 ± 0.28	0.393
	2	4.62 ± 1.10	4.69 ± 1.02	5.00 ± 0.00	0.232	4.59 ± 1.04	4.65 ± 1.02	4.88 ± 0.33	0.416
	3	4.65 ± 1.08	4.70 ± 1.01	5.00 ± 0.00	0.289	4.67 ± 1.00	4.70 ± 0.94	5.00 ± 0.00	0.259
	4	4.64 ± 1.08	4.72 ± 1.00	5.00 ± 0.00	0.271	4.71 ± 0.99	4.70 ± 1.01	5.00 ± 0.00	0.332
	Sub total	4.65 ± 1.07 ^b^	4.70 ± 1.01 ^b^	5.00 ± 0.00 ^a^	0.006 **	4.64 ± 1.01 ^b^	4.67 ± 0.99 ^b^	4.95 ± 0.22 ^a^	0.013 *
Ingredient control	1	4.56 ± 1.10	4.65 ± 1.02	4.88 ± 0.44	0.386	4.55 ± 1.05	4.61 ± 0.98	4.76 ± 0.52	0.640
	2	4.63 ± 1.08	4.67 ± 1.03	4.96 ± 0.20	0.331	4.64 ± 1.03	4.70 ± 1.01	4.96 ± 0.20	0.353
	3	4.63 ± 1.08	4.65 ± 1.02	4.96 ± 0.20	0.316	4.67 ± 1.00	4.65 ± 1.02	4.96 ± 0.20	0.339
	4	4.67 ± 1.08	4.72 ± 1.00	5.00 ± 0.00	0.318	4.72 ± 0.99	4.70 ± 1.01	5.00 ± 0.00	0.348
	5	4.65 ± 1.08	4.70 ± 1.01	5.00 ± 0.00	0.303	4.69 ± 1.00	4.67 ± 1.01	5.00 ± 0.00	0.295
	Sub total	4.63 ± 1.07 ^b^	4.68 ± 1.01 ^b^	4.96 ± 0.24 ^a^	0.004 **	4.65 ± 1.01 ^b^	4.67 ± 1.00 ^b^	4.94 ± 0.28 ^a^	0.001 **
Temperature control of food	1	4.64 ± 1.08	4.65 ± 1.02	5.00 ± 0.00	0.249	4.51 ± 1.09	4.61 ± 1.04	5.00 ± 0.00	0.100
	2	4.65 ± 1.08	4.72 ± 1.00	5.00 ± 0.00	0.294	4.71 ± 0.99	4.72 ± 1.00	5.00 ± 0.00	0.351
	3	4.56 ± 1.16	4.71 ± 1.01	5.00 ± 0.00	0.172	4.18 ± 1.33	4.28 ± 1.33	4.84 ± 0.55	0.075
	Sub total	4.63 ± 1.10 ^b^	4.69 ± 1.00 ^b^	5.00 ± 0.00 ^a^	0.015 *	4.49 ± 1.14 ^b^	4.54 ± 1.14 ^b^	4.95 ± 0.32 ^a^	0.004 **
Equipment and utensil sanitation	1	4.55 ± 1.06	4.63 ± 1.02	4.92 ± 0.28	0.254	4.49 ± 1.05	4.59 ± 1.02	4.72 ± 0.54	0.568
	2	4.64 ± 1.08	4.67 ± 1.01	4.96 ± 0.20	0.349	4.67 ± 1.00	4.65 ± 1.02	4.96 ± 0.20	0.339
	3	4.64 ± 1.08	4.72 ± 1.00	5.00 ± 0.00	0.271	4.68 ± 1.00	4.72 ± 1.00	5.00 ± 0.00	0.307
	Sub total	4.62 ± 1.07 ^b^	4.67 ± 1.00 ^b^	4.96 ± 0.20 ^a^	0.025 *	4.61 ± 1.01	4.65 ± 1.01	4.89 ± 0.35	0.074
Total		4.63 ± 1.07 ^b^	4.69 ± 1.00 ^b^	4.98 ± 0.16 ^a^	<0.001 ***	4.61 ± 1.04	4.64 ± 1.02	4.93 ± 0.29	<0.001 ^***^

Note: * *p* < 0.05, ** *p* < 0.001, *** *p* < 0.001 and superscript letters are significantly different at 5% significant level by Duncan’s multiple range test.

**Table 3:** Importance and performance of knowledge related to sanitation at childcare centers in Chungju (n = 150) by type of center.

Questions		Type and Score									
		Importance (Mean ± SD)				*p*-Value	Performance (Mean ± SD)				*p*-Value
		National /Public	Private	Home Type	Others		National /Public	Private	Home Type	Others	
Personal hygiene	1	4.04 ± 1.69 ^b^	4.82 ± 0.81 ^a^	4.85 ± 0.77 ^a^	5.00 ± 0.00	0.002 **	4.00 ± 1.64 ^b^	4.79 ± 0.82 ^a^	4.85 ± 0.46 ^a^	4.77 ± 0.53 ^a^	0.003 **
	2	4.00 ± 1.69 ^b^	4.78 ± 0.84 ^a^	4.81 ± 0.79 ^a^	4.95 ± 0.21	0.003 **	4.04 ± 1.66 ^b^	4.75 ± 0.85 ^a^	4.74 ± 0.53 ^a^	4.82 ± 0.39 ^a^	0.011 *
	3	4.13 ± 1.69 ^b^	4.78 ± 0.82 ^a^	4.85 ± 0.77 ^a^	4.95 ± 0.21 ^a^	0.014 *	4.09 ± 1.59 ^b^	4.78 ± 0.82 ^a^	4.96 ± 0.19 ^a^	4.86 ± 0.35 ^a^	0.002 **
	4	4.13 ± 1.69 ^b^	4.78 ± 0.82 ^a^	4.85 ± 0.77 ^a^	4.95 ± 0.21 ^a^	0.014 *	4.09 ± 1.68 ^b^	4.79 ± 0.82 ^a^	5.00 ± 0.00 ^a^	4.91 ± 0.29 ^a^	0.002 **
	Sub total	4.08 ± 1.66 ^b^	4.79 ± 0.82 ^a^	4.84 ± 0.76 ^a^	4.97 ± 0.01 ^a^	<0.001 ***	4.05 ± 1.62 ^b^	4.78 ± 0.82 ^a^	4.89 ± 0.37 ^a^	4.84 ± 0.40 ^a^	<0.001 ***
Ingredient control	1	4.04 ± 1.66 ^b^	4.75 ± 0.85 ^a^	4.74 ± 0.81 ^a^	4.72 ± 0.55 ^a^	0.022 *	4.04 ± 1.58 ^b^	4.70 ± 0.88 ^a^	4.78 ± 0.51 ^a^	4.55 ± 0.67 ^a^	0.024 *
	2	4.00 ± 1.68 ^b^	4.79 ± 0.82 ^a^	4.78 ± 0.80 ^a^	4.95 ± 0.21 ^a^	0.003 **	4.00 ± 1.69 ^b^	4.81 ± 0.83 ^a^	4.81 ± 0.48 ^a^	4.86 ± 0.35 ^a^	0.003 **
	3	3.96 ± 1.64 ^b^	4.79 ± 0.82 ^a^	4.85 ± 0.77 ^a^	4.86 ± 0.35 ^a^	0.001 **	3.96 ± 1.64 ^b^	4.81 ± 0.81 ^a^	4.93 ± 0.27 ^a^	4.86 ± 0.35 ^a^	<0.001 ***
	4	4.09 ± 1.68 ^b^	4.82 ± 0.81 ^a^	4.85 ± 0.77 ^a^	4.95 ± 0.21 ^a^	0.006 **	4.09 ± 1.68 ^b^	4.82 ± 0.81 ^a^	5.00 ± 0.00 ^a^	4.91 ± 0.29 ^a^	0.001 **
	5	4.04 ± 1.66 ^b^	4.81 ± 0.81 ^a^	4.85 ± 0.78 ^a^	4.95 ± 0.21 ^a^	0.003 **	4.00 ± 1.65 ^b^	4.79 ± 0.82 ^a^	5.00 ± 0.00 ^a^	4.95 ± 0.21 ^a^	<0.001 ***
	Sub total	4.03 ± 1.64 ^b^	4.79 ± 0.81 ^a^	4.81 ± 0.78 ^a^	4.89 ± 0.34 ^a^	<0.001 ***	4.02 ± 1.62 ^b^	4.79 ± 0.82 ^a^	4.90 ± 0.34 ^a^	4.83 ± 0.43 ^a^	<0.001 ***
Temperature Control of food	1	3.96 ± 1.64 ^b^	4.79 ± 0.82 ^a^	4.85 ± 0.77 ^a^	4.86 ± 0.35 ^a^	0.001 **	4.00 ± 1.65 ^b^	4.70 ± 0.89 ^a^	4.78 ± 0.64 ^a^	4.72 ± 0.55 ^a^	0.016 ^*^
	2	4.09 ± 1.68 ^b^	4.82 ± 0.81 ^a^	4.85 ± 0.77 ^a^	4.91 ± 0.29 ^a^	0.007 **	4.09 ± 1.68 ^b^	4.82 ± 0.81 ^a^	5.00 ± 0.00 ^a^	4.91 ± 0.29 ^a^	0.001 **
	3	4.13 ± 1.69 ^b^	4.79 ± 0.83 ^a^	4.78 ± 0.85 ^a^	4.83 ± 0.51 ^a^	0.044 *	3.73 ± 1.78 ^b^	4.56 ± 1.05 ^a^	4.35 ± 1.11 ^a^	4.33 ± 1.14 ^a^	0.059
	Sub total	4.06 ± 1.64 ^b^	4.80 ± 0.81 ^a^	4.83 ± 0.78 ^a^	4.90 ± 0.35 ^a^	<0.001 ***	3.94 ± 1.68 ^b^	4.70 ± 0.92 ^a^	4.73 ± 0.75 ^a^	4.68 ± 0.74 ^a^	<0.001 ^***^
Equipment and utensil sanitation	1	4.04 ± 1.58 ^b^	4.71 ± 0.84 ^a^	4.74 ± 0.81 ^a^	4.82 ± 0.50 ^a^	0.018 *	4.00 ± 1.90 ^b^	4.66 ± 0.89 ^a^	4.70 ± 0.67 ^a^	4.55 ± 0.60 ^a^	0.032 *
	2	4.04 ± 1.66 ^b^	4.79 ± 0.82 ^a^	4.81 ± 0.79 ^a^	4.91 ± 0.29 ^a^	0.005 **	4.00 ± 1.65 ^b^	4.79 ± 0.82 ^a^	4.93 ± 0.27 ^a^	4.86 ± 0.35 ^a^	0.001 **
	3	4.09 ± 1.68 ^b^	4.82 ± 0.81 ^a^	4.85 ± 0.77 ^a^	4.86 ± 0.35 ^a^	0.009 **	4.09 ± 1.68 ^b^	4.84 ± 0.80 ^a^	4.93 ± 0.27 ^a^	4.86 ± 0.35 ^a^	0.003 **
	Sub total	4.06 ± 1.62 ^b^	4.78 ± 0.82 ^a^	4.80 ± 0.78 ^a^	4.86 ± 0.39 ^a^	<0.001 ***	4.03 ± 1.62 ^b^	4.76 ± 0.83 ^a^	4.85 ± 0.45 ^a^	4.76 ± 0.47 ^a^	<0.001 ***
Total		4.05 ± 1.63 ^b^	4.79 ± 0.81 ^a^	4.82 ± 0.77 ^a^	4.91 ± 0.32 ^a^	<0.001 ***	4.01 ± 1.62 ^b^	4.76 ± 0.84 ^a^	4.86 ± 0.48 ^a^	4.79 ± 0.50 ^a^	<0.001 ***

Note: * *p* < 0.05, ** *p* < 0.001, *** *p* < 0.001 and superscript letters are significantly different at 5% significant level by Duncan’s multiple range test.

**Table 4:** Importance and performance of knowledge related to sanitation at childcare centers in Chungju (n = 150) by enrolled children.

Questions		Enrolled Children and Score					
		Importance (Mean ± SD)		*p*-Value	Performance (Mean ± SD)		*p*-Value
		<50	≥50		<50	≥50	
Personal hygiene	1	4.91 ± 0.57	4.33 ± 1.48	0.014 *	4.86 ± 0.51	4.29 ± 1.47	0.014 *
	2	4.86 ± 0.62	4.33 ± 1.46	0.024 *	4.78 ± 0.58	4.33 ± 1.46	0.052
	3	4.88 ± 0.59	4.36 ± 1.46	0.024 *	4.89 ± 0.47	4.33 ± 1.41	0.013 *
	4	4.89 ± 0.58	4.33 ± 1.46	0.017 *	4.91 ± 0.45	4.36 ± 1.46	0.016 *
	Sub total	4.89 ± 0.59	4.34 ± 1.45	<0.001 ***	4.86 ± 0.50	4.33 ± 1.44	<0.001 ***
Ingredient control	1	4.78 ± 0.67	4.31 ± 1.46	0.043 *	4.71 ± 0.65	4.31 ± 1.41	0.074
	2	4.87 ± 0.59	4.29 ± 1.47	0.013 *	4.89 ± 0.49	4.27 ± 1.47	0.008 **
	3	4.86 ± 0.60	4.29 ± 1.46	0.014 ^*^	4.90 ± 0.46	4.27 ± 1.45	0.006 **
	4	4.90 ± 0.57	4.36 ± 1.46	0.019 *	4.94 ± 0.42	4.33 ± 1.46	0.009 **
	5	4.90 ± 0.58	4.31 ± 1.46	0.012 **	4.93 ± 0.43	4.29 ± 1.46	0.006 **
	Sub total	4.86 ± 0.60	4.31 ± 1.45	<0.001 ***	4.88 ± 0.50	4.29 ± 1.44	<0.001 ***
Temperature control of food	1	4.88 ± 0.59	4.29 ± 1.46	0.011 *	4.79 ± 0.62	4.24 ± 1.46	0.019 *
	2	4.91 ± 0.57	4.31 ± 1.46	0.010 *	4.95 ± 0.41	4.31 ± 1.46	0.006 **
	3	4.85 ± 0.65	4.31 ± 1.46	0.036 *	4.36 ± 1.12	4.36 ± 1.46	0.983
	Sub total	4.88 ± 0.60	4.32 ± 1.45	<0.001 ***	4.72 ± 0.79	4.30 ± 1.45	0.002 ***
Equipment and utensil sanitation	1	4.77 ± 0.66	4.31 ± 1.41	0.041 *	4.66 ± 0.68	4.27 ± 1.42	0.080
	2	4.88 ± 0.59	4.29 ± 1.46	0.011 *	4.89 ± 0.47	4.29 ± 1.46	0.009 **
	3	4.89 ± 0.58	4.33 ± 1.46	0.017 *	4.92 ± 0.44	4.33 ± 1.46	0.011 *
	Sub total	4.85 ± 0.61	4.31 ± 1.43	<0.001 ***	4.83 ± 0.55	4.30 ± 1.44	<0.001 ***
Total		4.87 ± 0.60	4.32 ± 1.44	<0.001 ***	4.83 ± 0.58	4.31 ± 1.44	<0.001 ***

Note: * *p* < 0.05, ** *p* < 0.001, *** *p* < 0.001.

## Data Availability

Not applicable.

## Supplementary Materials

The following supporting information can be downloaded at: https://www.mdpi.com/article/10.3390/ijerph19159668/s1, Table S1: General characteristics of respondents & childcare centers in Chungju (n = 150).
