# Supplementary material for: A Study of Food Safety Knowledge for Sustainable Foodservice Management of Childcare Centers in South Korea Using Importance–Performance Analysis
Source: Int J Environ Res Public Health. 2022 Aug 5;19(15):9668. doi: 10.3390/ijerph19159668 (PMC9368346; doi:10.3390/ijerph19159668)
Supplement: Supplementary file 1 [file ijerph-19-09668-s001.zip › ijerph-1831683-supplementary.pdf]

Supplementary Table S1. General characteristics of respondents & childcare centers in Chungju (n = 150).

**Characteristics**			**Frequency (N)**	**Percent (%)**
**Respondents**	**Age**	<40s	50	33.4
		50s	64	42.7
		≧60	35	23.3
	**Working duration (yrs)**	<5	78	52.3
		5-10	46	30.9
		>10	25	16.8
	**Certificate**	Yes	143	95.3
		No	7	4.7
	**Daily number of food serving**	Breakfast	13	8.7
		Mid morning snack	124	82.7
		Lunch	133	88.7
		Afternoon snack	122	81.3
		Dinner	22	14.7
	**Daily working hours (h)**	<4	29	19.3
		4-8	112	74.7
		>8	8	5.3
**Childcare centers**	**Types of childcare centers**	National/public	23	15.3
		Private	73	48.7
		Home type	27	18.0
		Others	22	14.7
	**Number of enrolled children**	<50	101	69.2
		≧50	45	30.8
